# The Scientific American

**Published:** 1880-12

**Authors:** 


### EDITORIAL.

### THE SCIENTIFIC AMERICAN.

This is a large *first-class Weekly Newspaper*, containing sixteen pages, very finely printed and profusely illustrated with splendid ngravings, representing the newest Inventions, and the most recent advances in the Arts and Sciences, including New and Interesting Facts in Agriculture, Horticulture, the Home, Health, Medical Progress, Social Science, Natural History, Geology and Astronomy.

The most valuable, practicable papers by eminent writers in all departments of Science, will be found in the Scientific American This paper has grown in importance and value annually, during the thirty-six years of its existence.

The subscription is $3.20 per year, including postage.

The SCIENTIFIC AMERICAN SUPPLEMENT, also published weekly, is also a very valuable paper, in some respects more so than the "*Scientific American.*"

All who wish to keep well posted in the fields which they cultivate should have both of these papers. The Supplement is $5 per year, and the two will be sent to one address for $7. That amount of money can not be invested in any direction in which it will give a larger return.

Messrs. Munn & Co. are also solicitors of American and Foreign Patents, and have the advantage of a long, varied and extensive experience; in this work they have the largest establishment in the world.

Any person who has made a new discovery or invention can ascertain, *free of charge*, whether a patent can probably be obtained by writing to Munn & Co., New York. They, also, upon application, send *free* their Hand Book which gives much valuable information on Patent laws, Patent Grants, Trade marks, their cost and how procured, etc.

Every Dentist who at all desires to know of the state and progress of general science should have both *the* "SCIENTIFC AMERICAN" and the "SUPPLEMENT."

